# Parallel Genome‐Wide CRISPR Screens Reveal SORL1 and ZFYVE19 as Sequential Host Determinants of *Salmonella* Infection

**DOI:** 10.1002/advs.202515042

**Published:** 2025-11-23

**Authors:** Sehee Yun, Seoyeon Kim, Seonggyu Kim, Minsoo Noh, Dae‐Kyum Kim, Eun‐Jin Lee, Hunsang Lee

**Affiliations:** ^1^ Department of Life Sciences School of Life Sciences and Biotechnology Korea University Seoul 02841 Republic of Korea; ^2^ National Research Laboratory for Convergence Degradation Biology Korea University Seoul 02841 Republic of Korea; ^3^ Department of Internal Medicine and Laboratory of Genomics and Translational Medicine Gachon University College of Medicine Incheon 21565 Republic of Korea; ^4^ Division of Thoracic and Upper Gastrointestinal Surgery Department of Surgery Faculty of Medicine and Health Sciences McGill University Montreal H4A 3J1 Canada; ^5^ Cancer Research Program Research Institute of McGill University Health Centre Montreal H3H 2R9 Canada

**Keywords:** CRISPR screen, host‐directed therapy, host‐pathogen interactions, *Salmonella*, SORL1, ZFYVE19

## Abstract

*Salmonella enterica*, a major cause of gastroenteritis and typhoid fever, hijacks host machinery to invade cells, and replicate within a specialized niche. While some host factors are known, a comprehensive, temporally‐resolved understanding of the host‐pathogen interface has been hindered by a lack of suitable genome‐wide methodologies. To address this, a parallel CRISPR screening platform is developed to identify host determinants for distinct infection stages. An invasion screen captured factors for bacterial entry, while a fitness screen identified factors governing long‐term survival. The screens reveal a temporal switch in host dependency, from endosomal trafficking in early infection to cell cycle and DNA damage response pathways governing host cell fitness in long‐term infection. Notably, the approach uncovers two novel host factors with stage‐specific roles, SORL1 as a mediator of bacterial invasion and ZFYVE19 as a factor supporting intracellular proliferation. Genetic disruption of SORL1 or ZFYVE19 validate these roles, leading to impaired invasion or replication, respectively. Importantly, antibody‐mediated blockade of SORL1 effectively prevented *Salmonella* entry, highlighting it as a novel host‐directed therapeutic target. Together, the screening strategy provides a powerful framework for the temporal dissection of host‐pathogen interactions, revealing novel biology and promising therapeutic targets.

## Graphical abstract

To distinguish how *Salmonella* invades cells vs how it survives long‐term, a parallel CRISPR screening platform is developed. This approach reveals the host proteins that the bacterium exploits at different stages of infection. The study identifies SORL1 as a novel host factor for invasion and demonstrates that blocking it with an antibody effectively prevents infection, revealing a novel host‐directed therapeutic strategy.

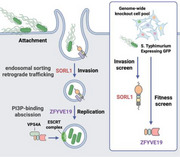

## 1 Introduction

As a Gram‐negative facultative intracellular pathogen, *Salmonella enterica* has evolved sophisticated strategies to invade host cells, evade immune responses, and establish a replicative niche, causing diseases such as gastroenteritis and typhoid fever.^[^ ^1^, ^2^ ^]^ Central to its lifecycle is the manipulation of host cellular machinery. During the invasion of non‐phagocytic cells, *Salmonella* utilizes a type III secretion system 1 (T3SS1) to trigger dramatic cytoskeletal rearrangements and membrane ruffling, regulated by host factors like Rho GTPases.^[^ ^3^, ^4^, ^5^, ^6^ ^]^ In professional phagocytes, internalization primarily occurs via classical phagocytosis.^[^ ^7^ ^]^ Following internalization, the bacterium resides within a specialized *Salmonella*‐containing vacuole (SCV), the biogenesis and stability of which depend on the host vesicular trafficking pathways, including Rab GTPases and the endosomal sorting complexes required for transport (ESCRT) machinery.^[^ ^8^, ^9^, ^10^, ^11^, ^12^, ^13^ ^]^ This intracellular lifestyle presents a difficult challenge for treatment, especially in the face of rising antimicrobial resistance.^[^ ^14^ ^]^

To deconstruct the complex host‐pathogen interactions governing these processes, unbiased, genome‐wide approaches have become valuable.^[^ ^15^ ^]^ Previous functional genomics studies, including RNAi and CRISPR‐based screens, have successfully identified hundreds of host factors involved in *Salmonella* pathogenesis.^[^ ^16^, ^17^, ^18^ ^]^ The identified factors include key components of autophagy and intracellular trafficking, which modulate intracellular *Salmonella* survival, as well as metabolic and cytoskeletal regulators involved in phagocyte invasion. These genetic screens, complemented by proteomics‐based methods, have built a valuable snapshot of the host‐effector interactome. For example, proteomic approaches like BioID have identified direct host partners of five *Salmonella* effectors, while a large‐scale affinity purification‐quantitative mass spectrometry (AP‐QMS) study mapped 446 protein‐protein interactions across 15 *Salmonella* effectors.^[^ ^19^, ^20^ ^]^

However, these studies are often limited by their design, typically capturing a static view of the infection. Bacterial pathogenesis is a dynamic, multi‐stage process, yet most screens do not provide temporal resolution, making it difficult to distinguish host factors required for initial invasion from those essential for subsequent intracellular replication and survival. Furthermore, a significant technical barrier, the toxicity from uncontrolled extracellular bacterial growth in long‐term co‐cultures, has largely prevented the systematic investigation of host factors that determine cell fitness over the full course of an infection.

To overcome these limitations, we engineered a dual‐component CRISPR screening platform. By adapting a culture method that permits the long‐term viability of infected host cells, we were able to execute two parallel screens, an invasion screen to identify host determinants of bacterial entry, and a fitness screen to uncover factors governing long‐term host cell survival. This strategy provides a temporally‐resolved perspective on *Salmonella* infection, allowing us to functionally separate the molecular requirements of distinct pathogenic stages.

## 2 Results

### 2.1 A Parallel Genome‐Wide CRISPR Screen Distinguishes Host Factors for *Salmonella* Invasion and Host Cell Fitness

**Figure 1:**
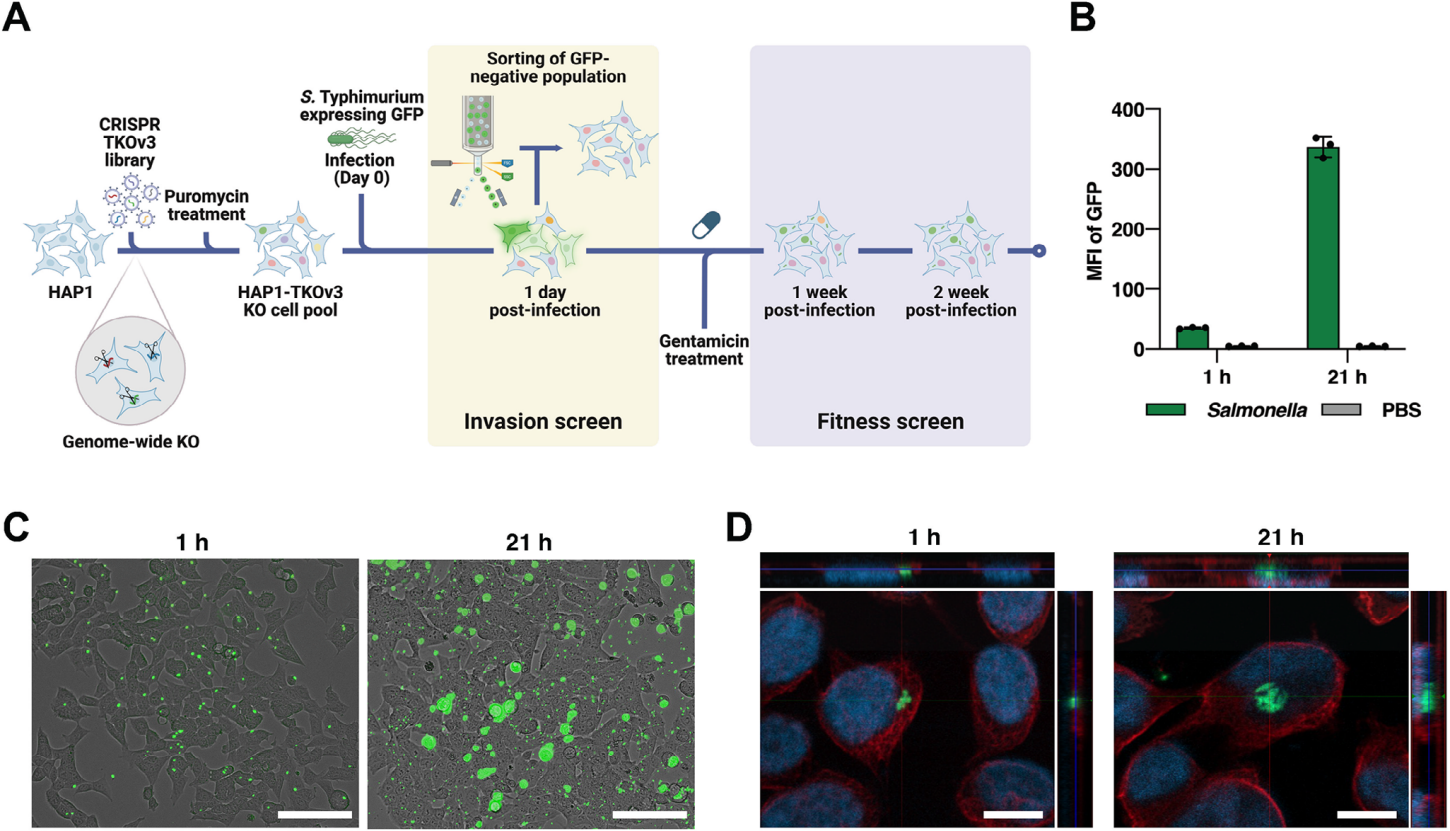
A parallel CRISPR screening platform for distinguishing host genes governing early and late stages of *Salmonella* infection. A) Schematic of the parallel CRISPR screening strategy in HAP1 cells. B) Flow cytometric quantification of intracellular bacterial load. Cells were infected at a MOI of 50, and the mean fluorescence intensity (MFI) of GFP was measured. Data are presented as mean ± SD from three independent experiments (n = 3). C) Representative fluorescence microscopy images of HAP1 cells infected with GFP‐expressing *Salmonella*. Following infection as in (B), images were acquired at 1 and 21 h post‐infection. Scale bar, 100 µm. D) Representative confocal fluorescence microscopy images of HAP1 cells infected with GFP‐expressing *Salmonella*. Cells were stained for nuclei (blue) and 𝛼‐tubulin (red). *Salmonella* are visualized by GFP (green). Scale bar, 10 µm.

To deconstruct the multi‐stage nature of *Salmonella* infection, we designed a parallel, genome‐wide CRISPR screening platform to functionally separate host factors required for initial invasion from those governing long‐term host cell survival post‐infection (**Figure** **1**A).^[^ ^21^ ^]^ While conventional screens have identified host factors, they often cannot resolve these distinct temporal phases.^[^ ^18^ ^]^

We first established a robust infection model using GFP‐expressing *Salmonella* in HAP1 cells, a near‐haploid human cell line highly amenable to genetic screening. At an optimized multiplicity of infection (MOI of 50), we observed successful bacterial invasion by 1 h post‐infection, which progressed to significant intracellular replication within expanding intracellular foci by 21 h (Figure 1B,C). To confirm the intracellular localization of *Salmonella* within HAP1 cells, we performed confocal microscopy with z‐stack imaging. Three‐dimensional reconstruction of the images revealed that GFP‐expressing *Salmonella* were enclosed within the 𝛼‐tubulin‐defined cytoplasm and underwent intracellular replication (Figure 1D).

Having established this model, we performed two parallel screens using the TKOv3 CRISPR library, which targets 18,053 host genes with an average of 4 gRNAs per gene. In the first arm, the invasion screen, we infected a library‐transduced HAP1 cell population and used FACS to isolate the GFP‐negative cells, those that successfully resisted bacterial entry. By comparing gRNA abundance in this resistant population to the control, we could identify host factors whose loss confers protection from invasion (Figure 1A, left panel).

In the second arm, the fitness screen, we sought to identify host factors essential for long‐term cell survival during chronic infection. Infected cells were cultured for 14 days in a gentamicin‐containing medium to eliminate extracellular bacteria and ensure host cell viability. During this period, cells lacking key tolerance factors are depleted from the population. By sequencing gRNAs from surviving cells at late time points (day 7 and day 14) and comparing them to non‐infected controls, we identified host genes required to survive a sustained *Salmonella* infection (Figure 1A, right panel).

### 2.2 The Invasion Screen Identifies the Sorting Receptor SORL1 and Endosomal Trafficking Pathways as Key Factors for *Salmonella* Entry

**Figure 2:**
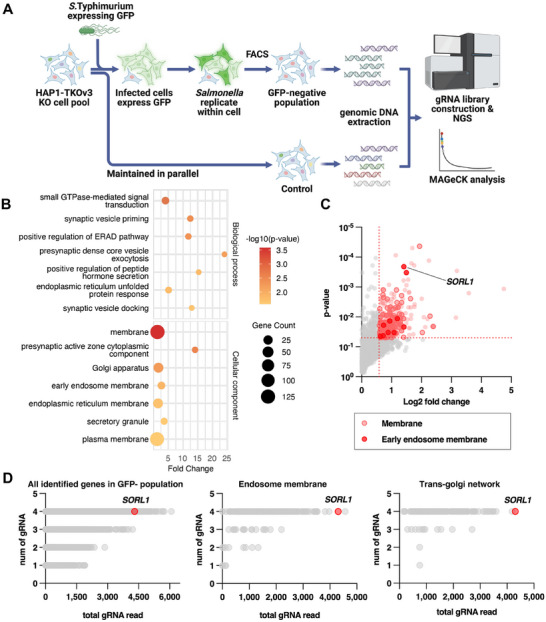
The CRISPR invasion screen identifies endosomal trafficking pathways and the novel host factor SORL1. A) Schematic of the CRISPR invasion screen. B) GO enrichment analysis of the 451 candidate genes identified in (A). The dot's size corresponds to the number of genes in each GO term, and the color represents the *p*‐value. C) Volcano plot of gene hits from the invasion screen. The log2​ fold change of gRNA abundance is plotted against statistical significance (*p*‐value). Genes meeting the hit threshold (log2​ fold change >0.58, *p*‐value <0.05) are highlighted in pink. Genes encoding membrane proteins are marked with a red border, and those specifically localized to the early endosome are indicated by an additional red dot. D) Genes identified in (A) are plotted based on the number of unique sgRNAs (*y*‐axis) and total sgRNA reads (*x‐*axis). A total of 271 genes identified in (A) are associated with the endosome membrane, with 161 annotated as TGN‐associated genes. Top hit, SORL1, is marked in red.

Analysis of the invasion screen, which identified host genes whose loss confers resistance to *Salmonella* uptake, revealed 451 candidate factors (log2 fold change >0.58, *p*‐value < 0.05) (**Figure** **2**A). We validated the biological relevance of this dataset in several ways. First, Gene Ontology (GO) enrichment analysis of these hits confirmed a strong overrepresentation of pathways involved in vesicle formation, transport, and recycling (Figure 2B), consistent with the known mechanisms of *Salmonella* invasion.^[^ ^22^, ^23^, ^24^ ^]^ Second, a substantial portion of these candidate genes (133 of 451) encoded membrane proteins highly enriched for functions in the endocytic and secretory pathways, again confirming the screen's validity (Figure 2C).^[^ ^25^ ^]^ Finally, to validate this hit list, we first compared it against other high‐throughput datasets, including a previously published CRISPR screen^[^ ^18^ ^]^ and an AP‐QMS dataset,^[^ ^20^ ^]^ which revealed several overlapping candidates. In addition to this large‐scale comparison, a manual literature review confirmed that our screen also successfully recovered numerous genes independently reported in single‐gene studies to be involved in *Salmonella* or other bacterial infections (Figure S1, Supporting Information).

Beyond these known pathways, our screen uncovered novel candidates. Our hypothesis was that factors mediating the invasion step would primarily be membrane‐associated proteins involved in the endocytic pathway. We first filtered our 451 significant hits (Figure 2C) for genes with a membrane protein annotation, which greatly narrowed the list. We then specifically analyzed this subset for proteins localized to the endosomal system, as this is the key pathway *Salmonella* is known to hijack for entry and SCV maturation. When this stringent, biologically driven filter was applied, the Sortilin‐Related Receptor 1 (SORL1) emerged as the top‐ranking candidate associated with the early endosome membrane (Figure 2D). SORL1 is a type‐I transmembrane receptor reported to regulate cargo sorting between the trans‐Golgi Network (TGN) and endosomes, a function that has been primarily studied in neurobiology particularly in the context of Alzheimer's disease.^[^ ^26^, ^27^ ^]^ Although this trafficking role provides a strong biological rationale for its potential involvement, SORL1 has not previously been implicated in bacterial pathogenesis. Our findings therefore identify SORL1 as a novel host factor for *Salmonella* invasion.

### 2.3 Functional Validation Reveals SORL1 is Required for *Salmonella* Invasion but Not for Subsequent Intracellular Replication

**Figure 3:**
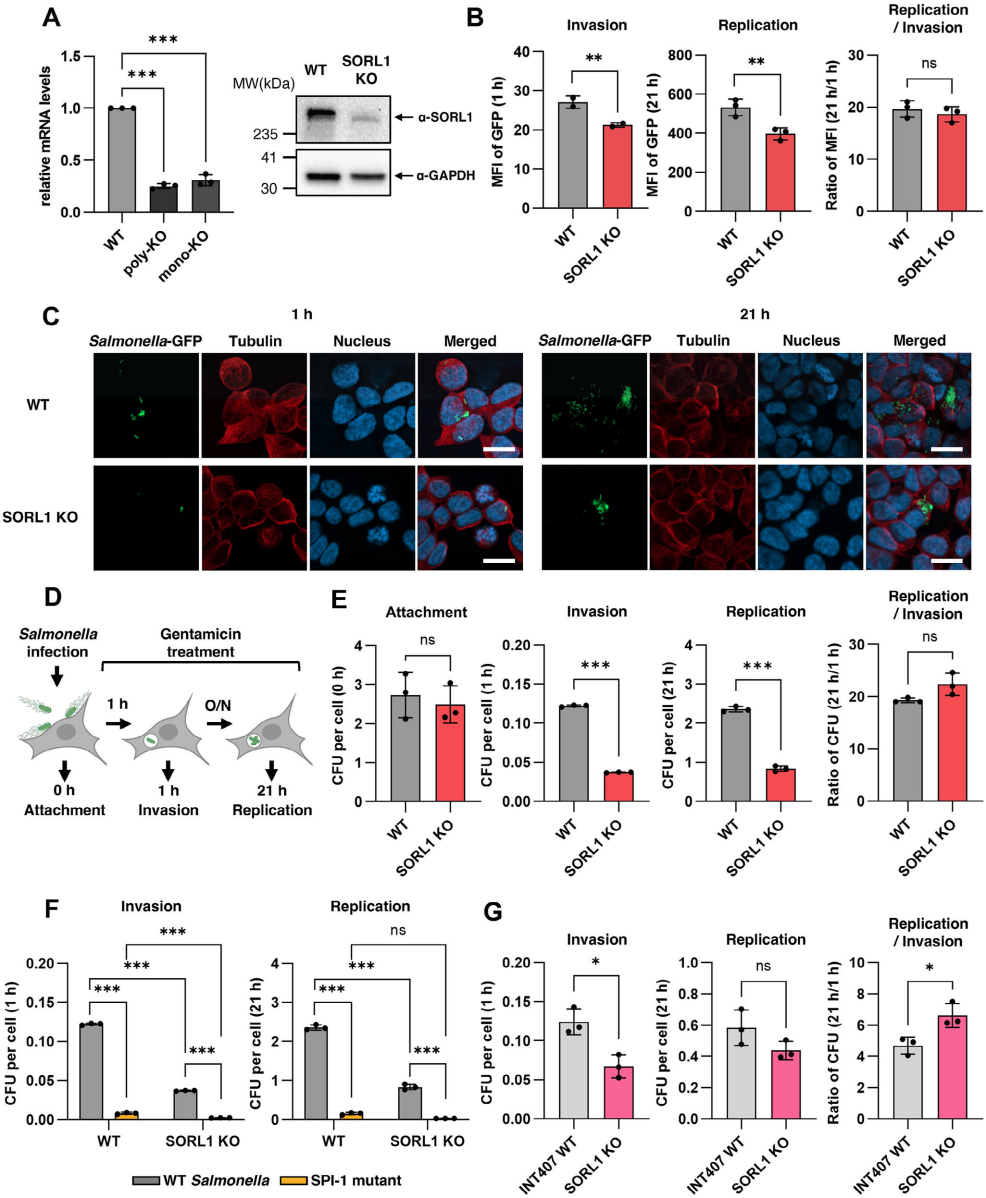
Functional validation reveals SORL1 is essential for *Salmonella* invasion. A) Validation of SORL1 KO in HAP1 cell lines. (Left) qRT‐PCR analysis confirming SORL1 transcript depletion in polyclonal (poly‐KO) and monoclonal (mono‐KO) lines relative to the parental WT control. *p* value ^***^; <0.001 (Right) western blot analysis showing the corresponding depletion of SORL1 protein in the KO cell lines. B) Flow cytometric analysis of bacterial load in HAP1 WT and SORL1 KO cells at 1 h (invasion) and 21 h (replication) post‐infection with GFP‐expressing *Salmonella*. The ratio of MFI at 21 to 1 h was also calculated as a metric for intracellular replication. Data represent mean ± SD (n = 3). *p* value ns; ≥0.5 and ^**^; <0.01. C) Representative confocal microscopy images of infected HAP1 WT and SORL1 KO cells at the indicated times. *Salmonella* are visualized by GFP (green). Cells were stained for 𝛼‐tubulin (red) and nuclei (blue). Merged images show the localization of *Salmonella* inside HAP1 cells. Scale bar, 20 µm. D) Schematic of intracellular survival assay. E) Quantification of bacterial attachment, invasion, and replication in HAP1 WT and SORL1 KO cells from the intracellular survival assay measured by CFUs. Fold attachment, invasion, and replication represent [number of bacteria at the indicated time/ number of HAP1 cells]. Replication ratio represents [number of bacteria at 21 h/ number of bacteria at 1 h]. Data represent mean ± SD (n = 3). *p* value ns; ≥0.5 and ^***^; <0.001. F) Quantification of bacterial invasion and replication of *Salmonella* WT or SPI‐1 mutant in HAP1 WT and SORL1 KO cells from the intracellular survival assay measured by CFUs. Data represent mean ± SD (n = 3). *p* value ns; ≥0.5 and ^***^; <0.001. G) Quantification of bacterial invasion and replication in INT407 WT and SORL1 KO cells from the intracellular survival assay measured by CFUs. Data represent mean ± SD (n = 3). *p* value ns; ≥0.5 and ^*^; <0.05.

To validate the role of SORL1, we generated polyclonal and monoclonal SORL1 knockout (KO) HAP1 cells using CRISPR‐Cas9 and confirmed the depletion of SORL1 transcripts via qRT‐PCR and western blot analyses (**Figure** **3**A). We then challenged these cells with GFP‐expressing *Salmonella* and assessed the intracellular bacterial load at 1 and 21 h post‐infection using flow cytometry. While SORL1 KO cells exhibited significantly reduced GFP mean fluorescence intensity (MFI) at both time points, the ratio of MFI at 21 to 1 h (a metric for intracellular replication) did not differ from wild‐type (WT) cells (Figure 3B). This indicates that SORL1 is required for the initial invasion step but has minimal impact on subsequent bacterial replication once inside the cell.

Confocal microscopy confirmed a significant reduction in the number of intracellular bacteria in SORL1 KO cells at both time points, though their perinuclear localization was unaltered (Figure 3C, Figure S2, Supporting Information). To confirm the specificity of this phenotype, we tested a control KO cell line targeting NLRX1, a gene not identified in our screen. NLRX1 KO cells showed no defect in *Salmonella* invasion compared to WT controls, supporting that the observed phenotype is specific to SORL1 deletion (Figure S3, Supporting Information).

To quantitatively confirm these findings with an orthogonal method, we employed a gentamicin protection assay, which only measures viable intracellular bacteria (Figure 3D). While the loss of SORL1 had no effect on the initial attachment of *Salmonella* to the cell surface, it caused a significant reduction in the number of intracellular bacteria as quantified by colony‐forming units (CFUs) at 1 h post‐infection (Figure 3E). While the total CFU in SORL1 KO cells at 21 h post‐infection appeared reduced, the fold increase relative to the CFU at 1 h post‐infection was comparable to that of the WT. Together, these data confirm that SORL1 is a main host factor controlling the initial invasion of *Salmonella*.

To place the SORL1's function within the known invasion pathway, we next sought to determine its relationship with the *Salmonella* Pathogenicity Island 1 (SPI‐1) T3SS.^[^ ^28^ ^]^ To this end, we generated a SPI‐1 deletion mutant strain (lacking the T3SS1) and performed invasion assays in both WT and SORL1 KO HAP1 cells. As expected, the SPI‐1 deletion mutant strain exhibited a profound invasion defect in WT cells (Figure 3F). Notably, invasion by this mutant was reduced even further in the SORL1 KO cells. This additive effect strongly suggests that SORL1 facilitates *Salmonella* invasion through a distinct mechanism that acts in parallel to, or independently of, the canonical SPI‐1 T3SS pathway.

Having established this key mechanistic insight, we next sought to confirm the physiological relevance of our findings beyond HAP1 cells by validating our primary hits in the intestinal epithelial cell line INT407. First, we generated SORL1 knockout INT407 cells and confirmed the loss of protein expression by western blot analysis (Figure S4, Supporting Information). We then performed gentamicin protection assays to assess *Salmonella* infection. Consistent with our previous results, the SORL1 KO INT407 cells exhibited a marked reduction in bacterial invasion but showed no significant defect in intracellular replication (Figure 3G). This confirms that SORL1's role in facilitating bacterial entry is conserved in a physiologically relevant epithelial context.

### 2.4 Antibody‐Mediated Blockade of SORL1 Suppresses *Salmonella* Infection

**Figure 4:**
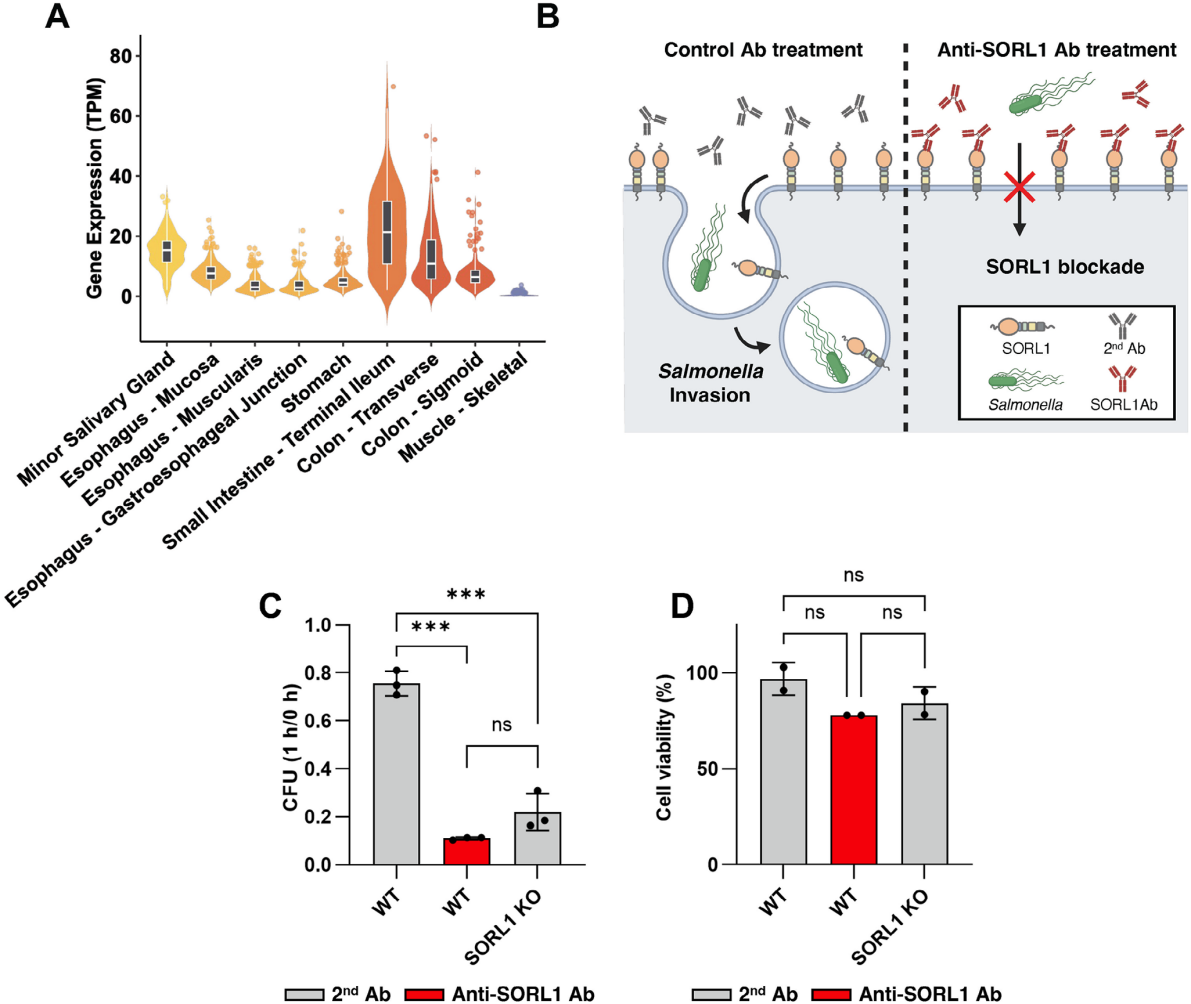
Antibody‐mediated inhibition of SORL1 is a potential host‐directed therapy for *Salmonella* infection. A) Violin plots illustrating SORL1 expression in various human gastrointestinal tissues and skeletal muscle (control). The *y*‐axis represents gene expression in Transcripts Per Million (TPM). Dots represent individual samples identified as outliers. The inner box plot displays the median and the interquartile range (IQR). B) Schematic of antibody‐mediated blockade of SORL1. C) Fold invasion of *Salmonella* within HAP1 WT or SORL1 KO cells treated with secondary antibody (2nd Ab control) or anti‐SORL1 antibody. Fold invasion represents [number of bacteria at 1 h / number of bacteria at 0 h]. Data represent mean ± SD (n = 3). *p* value ns; ≥0.5 and ^***^; <0.001. D) Determination of cell viability. The % of cell viability was calculated considering antibody‐untreated HAP1 WT as 100% and plotted. Data represent mean ± SD (n = 3). *p* value ns; ≥0.5.

Given that SORL1 is a novel invasion factor and an accessible cell surface receptor, we hypothesized it could serve as a host‐directed therapeutic target. Analysis of the Genotype‐Tissue Expression (GTEx) data confirmed that SORL1 is highly expressed throughout the human gastrointestinal tract, placing it at the primary site of *Salmonella* infection (**Figure** **4**A).

To test its therapeutic potential, we performed an antibody‐mediated inhibition experiment. As a topological feature, SORL1's extracellular domain makes it an ideal target for antibody‐based strategies. We treated WT HAP1 cells with an anti‐SORL1 antibody prior to infection and measured bacterial invasion (Figure 4B). The anti‐SORL1 antibody treatment significantly reduced *Salmonella* invasion to levels comparable to those observed in SORL1 KO cells, whereas a control antibody had no effect (Figure 4C). We also used a non‐specific control antibody targeting the Parathyroid Hormone 1 Receptor (PTH1R), a membrane protein abundantly expressed on HAP1 cells, and this confirmed the specificity of SORL1 inhibition in *Salmonella* infection (Figure S5A,B, Supporting Information). Cell viability assay confirmed that antibody treatment did not affect host cell viability, indicating that the difference in invasion efficiency was not due to cytotoxic effects (Figure 4D, Figure S5C, Supporting Information). These results demonstrate that functional inhibition of SORL1, either by genetic intervention or antibody blockade, substantially reduces bacterial entry and highlights SORL1 as a promising target for novel anti‐bacterial therapies.

### 2.5 A Long‐Term Fitness Screen Identifies Cell Cycle and DNA Damage Response Pathways as Mediators of Host Tolerance

**Figure 5:**
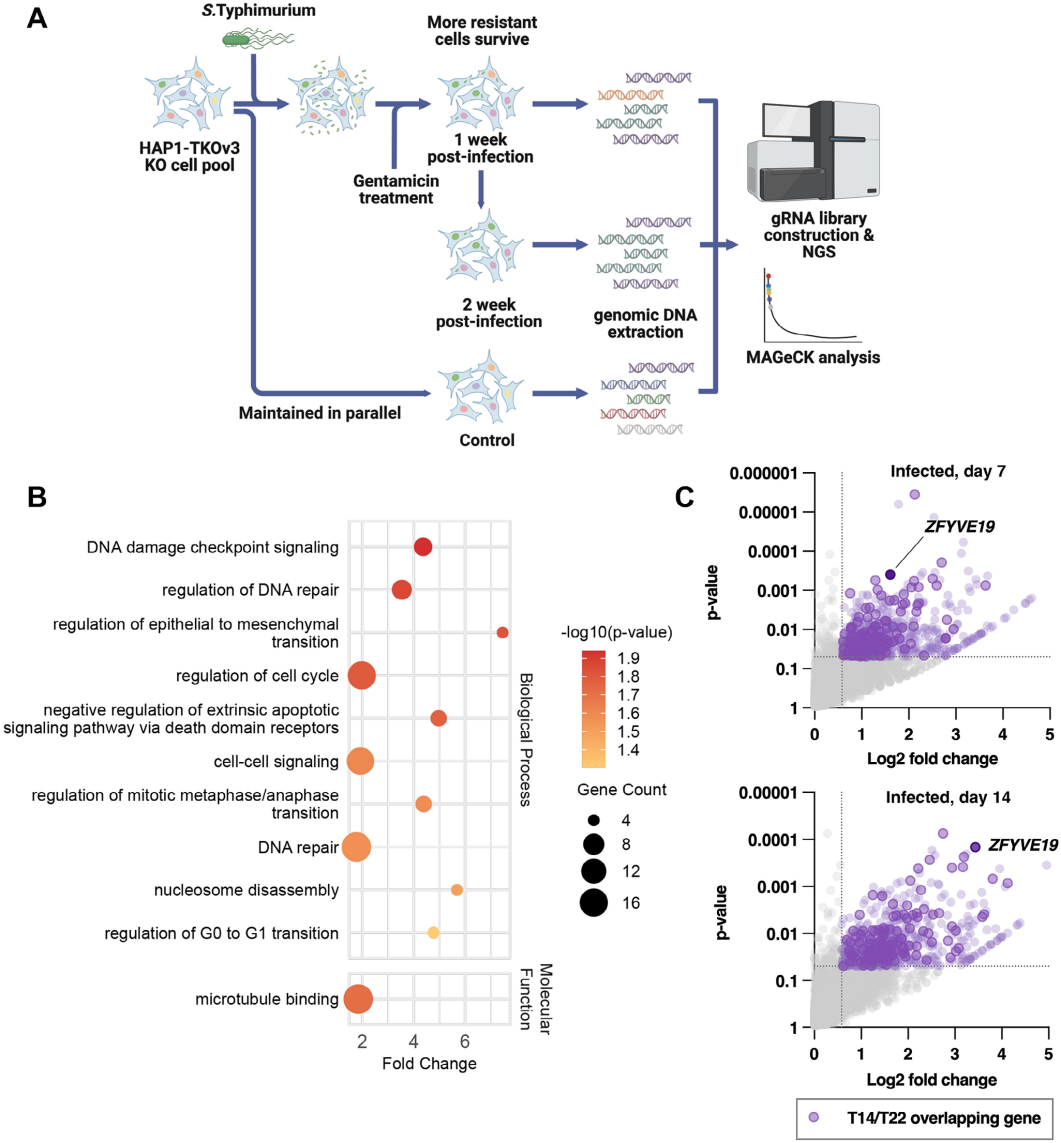
A fitness screen identifies host pathways regulating cell survival during *Salmonella* infection. A) Schematic representation of the long‐term cell fitness screen. B) GO enrichment analysis of enriched genes identified at the day 14 time point. Each dot represents an enriched biological term. The fold enrichment is plotted on the *x*‐axis, the *p* value is indicated by the dot's color, and the number of genes associated with each term is represented by the dot's size. C) Volcano plots showing genes whose knockout affects cell fitness at day 7 and day 14 post‐infection. Each plot displays the log2​ fold change of gRNA abundance compared to the uninfected control (*x*‐axis) vs the statistical significance (*y*‐axis). Positive hits (genes whose loss impacts fitness) are defined by a log2​ fold change >0.58 and a *p* value <0.05 and are highlighted in light purple. Genes identified as hits at both time points are marked with a purple border, and top‐ranking shared hits are indicated by a deep purple dot.

Analysis of our long‐term fitness screen, designed to identify host factors required for cell survival during prolonged infection, revealed distinct pathways governing host tolerance (**Figure** **5**A). GO analysis of genes depleted at day 14 of persistent *Salmonella* infection showed a significant overrepresentation of pathways involved in cell cycle regulation, DNA damage response, and microtubule organization (Figure 5B). This finding aligns with the known host responses to chronic infection, where pathogens disrupt cell cycle progression and induce DNA damage, thereby validating our screen's ability to identify key determinants of host tolerance.^[^ ^29^ ^]^

As further validation, we confirmed overlapping hits with candidates from a previously published CRISPR screen^[^ ^18^ ^]^ and an AP‐QMS dataset.^[^ ^20^ ^]^ A literature‐based review also confirmed the presence of numerous genes known to be involved in *Salmonella* or other bacterial infections, such as the known regulator CISH (Figure S6A, Supporting Information).^[^ ^30^ ^]^

In addition to the resistance factors that promote host survival, our fitness screen also identified a set of sensitizer genes, where loss‐of‐function rendered cells more susceptible to *Salmonella* infection. Notably, GO analysis of this sensitizer list revealed a significant enrichment for the autophagy pathway (Figure S6B, Supporting Information). This included the core component ATG5, a finding that aligns with previous literature demonstrating that ATG5‐deficient cells are more permissive to intracellular *Salmonella* growth.^[^ ^31^ ^]^

Based on this validation, we proceeded to analyze the resistant gene list to identify potential therapeutic targets. To this end, we focused on high‐confidence candidates that conferred a consistent survival advantage across both the Day 7 and Day 14 time points. This intersectional analysis identified Zinc Finger FYVE‐Type Containing 19 (ZFYVE19) as a top‐ranking candidate (Figure 5C).

Our selection of ZFYVE19 was based on its high statistical consistency (ranking #10 at Day 7 and #2 at Day 14) and its strong biological plausibility. The domain architecture of ZFYVE19 was particularly compelling. Its N‐terminal FYVE domain is known to bind Phosphatidylinositol 3‐phosphate (PI3P), a lipid *Salmonella* enriches on the SCV, while its C‐terminal domains are known to interact with the ESCRT complex, which is also important for SCV maturation. ZFYVE19 thus represented a novel potential link between these two key pathways. This combination of statistical consistency and pre‐existing biological relevance made ZFYVE19 our top candidate for in‐depth validation, particularly as it has a known role in centrosomal and endosomal trafficking but has not previously been linked to bacterial pathogenesis.

### 2.6 ZFYVE19 is Required for Intracellular *Salmonella* Replication via An Interaction with the ESCRT Machinery

**Figure 6:**
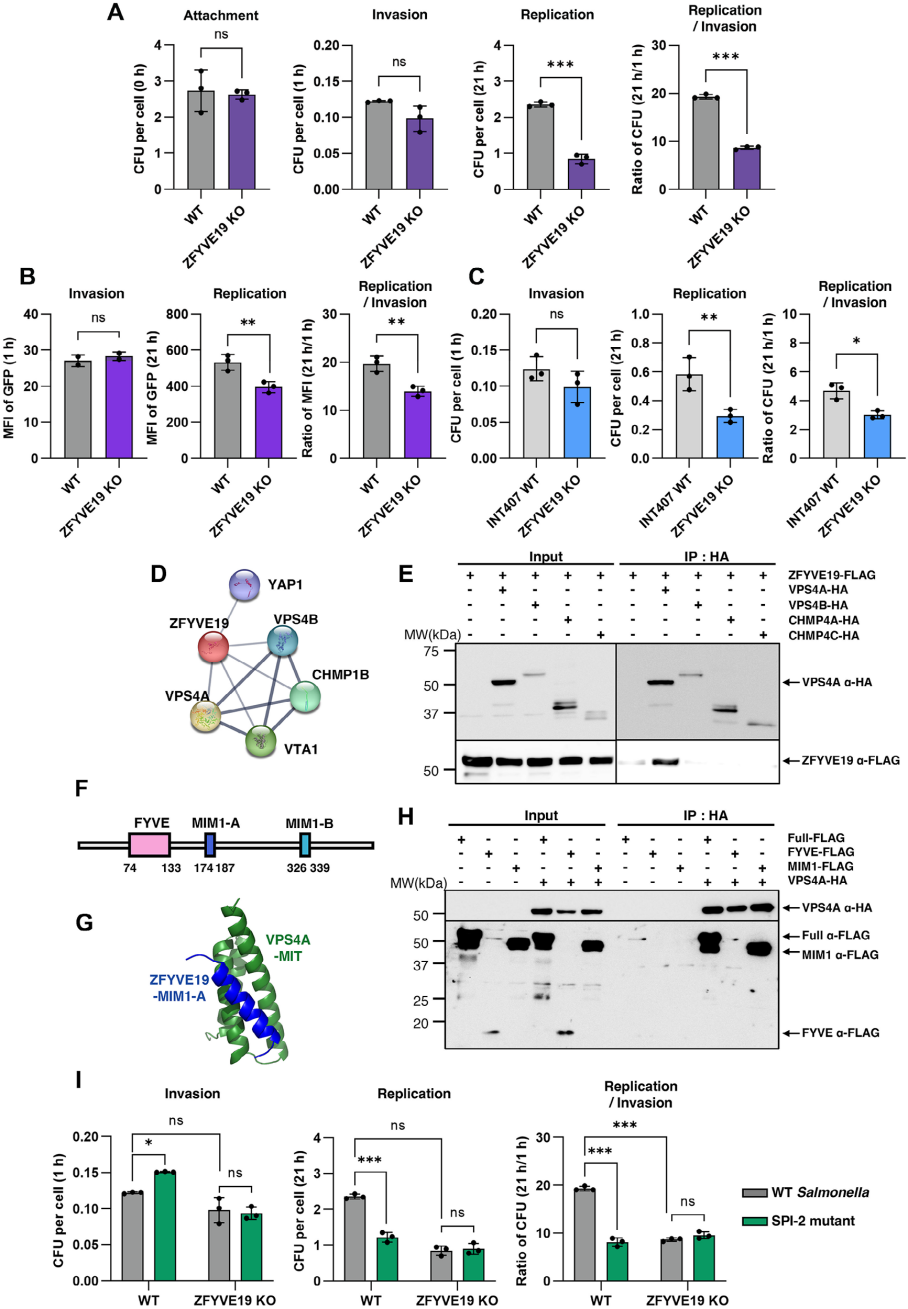
ZFYVE19 is required for intracellular replication of *Salmonella* and interacts with the ESCRT machinery. A) Quantification of bacterial attachment, invasion, and replication in HAP1 WT and ZFYVE19 KO cells from the intracellular survival assay measured by CFUs. Fold attachment, invasion, and replication represents [number of bacteria at the indicated time/ number of HAP1 cells]. Replication ratio represents [number of bacteria at 21 h/ number of bacteria at 1 h]. Data represent mean ± SD (n = 3). *p* value ns; ≥0.5 and ^***^; <0.001. B) Flow cytometric analysis of bacterial load in HAP1 WT and ZFYVE19 KO cells at 1 h (invasion) and 21 h (replication) post‐infection with GFP‐expressing *Salmonella*. The ratio of MFI at 21 to 1 h was also calculated as a metric for intracellular replication. Data represent mean ± SD (n = 3). *p* value ns; ≥0.5 and ^**^; <0.01. C) Quantification of bacterial invasion and replication in INT407 WT and ZFYVE19 KO cells from the intracellular survival assay measured by CFUs. Data represent mean ± SD (n = 3). *p* value ns; ≥0.5, ^*^; <0.05 and ^**^; <0.01. D) Protein‐protein interaction (PPI) network generated using STRING (v12.0) for candidate gene ZFYVE19. The minimum interaction score threshold was set to 0.55. E) Co‐immunoprecipitation of ZFYVE19 with VPS4A, VPS4B, CHMP4A and CHMP4C. HEK293T cells were transiently transfected with plasmids expressing FLAG‐tagged ZFYVE19 and HA‐tagged VPS4A, VPS4B, CHMP4A, and CHMP4C for 24 h. F) Domain structure of ZFYVE19 with FYVE and MIM1 domains. G) AlphaFold3‐assisted prediction of interaction between MIM1‐A domain of ZFYVE19 and MIT domain of VPS4A (iPTM=0.75, pTM=0.83). MIM1‐A domain of ZFYVE19 is shown in blue and MIT domain of VPS4A is shown in green. H) Co‐immunoprecipitation of VPS4A with ZFYVE19 (Full), ZFYVE19 FYVE domain (FYVE) and MIM1 domain (MIM1). HEK293T cells were transiently transfected with plasmids expressing HA‐tagged VPS4A and FLAG‐tagged ZFYVE19 or its respective domains. I) Quantification of bacterial invasion and replication of *Salmonella* WT or SPI‐2 mutant in HAP1 WT and ZFYVE19 KO cells from the intracellular survival assay measured by CFUs. Data represent mean ± SD (n = 3). *p* value ns; ≥0.5, ^*^; <0.05 and ^***^; <0.001.

To validate ZFYVE19's role, we generated ZFYVE19 KO HAP1 cells and confirmed efficient gene disruption by Tracking of Indels by Decomposition (TIDE) analysis (Figure S7, Supporting Information). We first sought to determine which stage of infection requires ZFYVE19. In contrast to the invasion factor SORL1, gentamicin protection assays revealed that loss of ZFYVE19 had no effect on bacterial attachment and invasion (**Figure** **6**A). However, intracellular replication was significantly reduced in ZFYVE19 KO cells by 21 h. Likewise, flow cytometry analysis revealed that the intracellular load was markedly reduced in ZFYVE19 KO cells (Figure 6B). A significantly lower 21 h/1 h MFI ratio in the KO cells confirmed this defect was due to impaired intracellular replication.

To test physiological relevance, we generated ZFYVE19 KO INT407 cells (Figure S7, Supporting Information). We challenged these cells with *Salmonella* and, in contrast to SORL1 KO cells, found they had no defect in initial bacterial invasion. However, by 21 h post‐infection, the ZFYVE19 KO cells contained significantly reduced bacterial CFUs compared to WT controls (Figure 6C).

Having validated from two cell lines that ZFYVE19 is a host factor specifically required for intracellular replication, we next investigated the underlying molecular mechanism, we investigated the ZFYVE19 protein interaction network. An analysis using the STRING database revealed strong interactions with multiple components of the ESCRT machinery, particularly the AAA‐ATPase VPS4 paralogs, VPS4A and VPS4B (Figure 6D). We experimentally confirmed this link via co‐immunoprecipitation, demonstrating a specific interaction between FLAG‐tagged ZFYVE19 and HA‐tagged VPS4A/B (Figure 6E). To map the basis of this interaction, we generated constructs expressing individual domains of ZFYVE19. Domain‐specific co‐IP assays revealed that the C‐terminal MIM1 (MIT‐interacting motif 1) domains were necessary and sufficient for binding VPS4, while the N‐terminal FYVE domain was not (Figure 6F–H). These findings indicate that ZFYVE19 engages the ESCRT machinery through its MIM1 domains, likely acting as a scaffold to recruit VPS4 to membrane compartments such as the SCV to support bacterial replication.

To genetically test our model, we next investigated whether ZFYVE19 functions in the same host pathway as the ESCRT complex, which is hijacked by SPI‐2 effectors.^[^ ^12^ ^]^ Previous work has shown that the replication defect of a SPI‐2 mutant is not further attenuated in ESCRT‐III (CHMP3) KO cells, demonstrating they function in the same pathway.^[^ ^13^ ^]^

Building on this precedent, we tested the infection efficiency of a SPI‐2 mutant in our ZFYVE19 KO cells. Consistent with our data in Figure 6A, replication of WT *Salmonella* was markedly impaired in ZFYVE19 KO cells (Figure 6I). In contrast, the SPI‐2 mutant strain exhibited a similar level of replication defect in both WT and ZFYVE19 KO cells.

Notably, the replication capacity of SPI‐2 T3SS mutant strain is unaffected when comparing it in WT and CHMP3 KO cells, highlighting ESCRT pathway.^[^ ^13^ ^]^ Building on this observation, we examined whether similar observations could hold true for ZFYVE19 KO. This non‐additive effect, where the loss of ZFYVE19 provides no additional defect to the SPI‐2 mutant, provides strong functional evidence that ZFYVE19 and the SPI‐2 T3SS operate within the same host pathway to promote efficient intracellular *Salmonella* replication.

Together, these data validate our key findings in an intestinal epithelial model, confirming that SORL1 is a stage‐specific factor for invasion, while ZFYVE19 is a distinct factor required for subsequent intracellular replication.

## 3 Discussion

In this study, we established a parallel genome‐wide CRISPR screening platform to overcome challenges of studying both short‐term invasion and long‐term host fitness. This dual‐component strategy allowed us to successfully deconstruct the *Salmonella* infection cycle into its distinct invasion and intracellular replication phases. This strategy not only identified novel host dependencies for each stage but also provides a powerful, broadly applicable platform for elucidating other complex host‐pathogen interactions. It is important to note that the factors identified in our screens exhibited a modest reduction in infection efficiency. This is likely attributable to the multifaceted nature of host‐microbe interactions, where many host factors, acting at different stages, contribute cooperatively or partially to the overall infection process. The genetic dependencies identified by our high‐throughput platform provide a rich resource of candidates for future validation in more physiologically relevant models, such as the advanced 3D co‐culture systems recently developed to study multicellular host‐pathogen interactions.^[^ ^32^ ^]^

Our invasion screen uncovered a previously unknown role for the multi‐domain sorting receptor SORL1 in bacterial pathogenesis. SORL1 contains both a VPS10 (vacuolar protein sorting 10) domain and an LDLR (low‐density lipoprotein receptor) domain, and it regulates intracellular trafficking via the TGN, early endosomes, and the plasma membrane.^[^ ^33^ ^]^ While SORL1's roles in neurodegenerative diseases (e.g., α‐synuclein clearance) and oncogenic receptor recycling are well‐documented,^[^ ^34^, ^35^ ^]^ its involvement in bacterial pathogenesis has not been previously reported. Here, we establish SORL1 as an essential host factor for the invasion phase of *Salmonella* infection. The precise molecular mechanism by which SORL1 facilitates *Salmonella* invasion, particularly given our finding that it acts independently of the canonical SPI‐1 T3SS pathway, remains a key question. A plausible hypothesis for future investigation is that SORL1 may regulate alternative host pathways, such as modulating the actin cytoskeleton or the activation of small GTPases like Rac1/Cdc42, which are also known to mediate bacterial uptake. This role is likely to be broadly relevant, as SORL1 is highly expressed in the gastrointestinal tract. Furthermore, its reported function in regulating the migration of phagocytic monocytes suggests that SORL1's importance may extend beyond epithelial cell invasion to also encompass interactions with professional phagocytes,^[^ ^36^ ^]^ further highlighting its significance in overall pathogenesis. The combination of its key role and its nature as an accessible cell‐surface receptor makes it a particularly attractive drug target, a concept validated by previous studies where the antibody targeting the SORL1 ectodomain has shown efficacy as an anti‐cancer agent.^[^ ^37^ ^]^ A key consideration for any anti‐invasion strategy is its utility in a clinical setting, where a patient would present with an already‐established infection. It is important to frame the infection not as a single event, but as a continuous dynamic cycle of invasion, replication, and cell‐to‐cell spread. Therefore, a therapeutic agent that blocks SORL1 would function to break this cycle of re‐invasion. By limiting the pathogen's ability to infect new host cells, such a treatment would reduce the overall bacterial burden, providing the host's immune system an advantage to clear the existing infection. This host‐directed approach is particularly promising as it would be separated from pathogen‐specific antibiotic resistance, offering a valuable strategy against multi drug resistant strains. This concept merits further investigation in pre‐clinical animal models of established infection.

The power of our platform to identify clinically translatable targets is further highlighted by our identification of NR2F6 as among the top resistance factors. This finding is particularly timely, as the therapeutic potential of targeting NR2F6 was independently validated in vivo in a study by Woelk et al.^[^ ^38^ ^]^ Beyond this, the biological significance of our fitness screen is validated by the rediscovery of several known regulators of *Salmonella* pathogenesis. For example, Cytokine Inducible SH2 Containing Protein (CISH) was identified as a high‐ranking candidate, a finding consistent with previous reports that Cish^−/−^ mice exhibit a significant reduction in bacterial burden.^[^ ^30^ ^]^ Our platform's ability to also rediscover canonical defense pathways, such as the ATG5‐mediated autophagy response in our sensitizer list, further validates the biological relevance of our dataset. This sensitizer list, in particular, represents a rich resource for future studies, although it would require further refinement, such as filtering against HAP1‐specific gene expression, to best prioritize candidates for future investigation.

In parallel, our long‐term fitness screen identified ZFYVE19 as a host factor specifically required for intracellular *Salmonella* replication. ZFYVE19 contains a FYVE domain that binds PI3P, a lipid enriched on endosomal membranes.^[^ ^39^ ^]^ It also harbors a MIM1 domain that enables interaction with VPS4, a key ATPase in the ESCRT pathway.^[^ ^40^, ^41^ ^]^ Our data support a molecular model in which ZFYVE19 acts as a molecular scaffold, recruited to the PI3P‐rich SCV membrane via its FYVE domain. Once localized, it engages the ESCRT machinery through its MIM1 domain by binding to the ATPase VPS4.^[^ ^40^ ^]^ Since *Salmonella* effectors like SopB are known to enrich PI3P on the SCV,^[^ ^42^ ^]^ and the ESCRT complex is essential for SCV membrane dynamics,^[^ ^13^ ^]^ ZFYVE19 emerges as a key, previously unrecognized link between these two pathways. ZFYVE19 is best characterized as a key checkpoint protein regulating membrane scission during cytokinesis.^[^ ^40^ ^]^ This raises the plausible hypothesis that it performs a similar scission role for the SCV. While this possibility cannot be completely excluded, our findings strongly support a distinct model. The ability of ZFYVE19 to bind PI3P, combined with its direct interaction with the ESCRT‐remodeling ATPase VPS4, suggests it primarily acts as a scaffold to modulate the SCV membrane in a manner that supports bacterial replication.

In summary, our dual CRISPR screening platform has successfully identified novel host factors governing distinct stages of *Salmonella* infection. The discoveries of SORL1 as an invasion factor and ZFYVE19 as a replication factor highlight how *Salmonella* hijacks distinct aspects of host endosomal and membrane trafficking pathways. In particular, SORL1 emerges as a promising candidate for therapeutic intervention. Targeting a fundamental host process like invasion offers a strategy that is distinct from pathogen‐specific antibiotic resistance. Furthermore, the parallel screening approach established here provides a powerful and broadly applicable framework for deconstructing the temporal infection dynamics of other challenging intracellular pathogens.

## 4 Experimental Section

### Bacterial and Cell Line Culture

Bacterial strains and plasmids used in this study are listed in Table S1 (Supporting Information). *S. enterica* serovar Typhimurium strains were derived from the wild‐type strain 14028s.^[^ ^43^ ^]^ DNA oligonucleotides are listed in Table S2 (Supporting Information). Bacteria were grown at 37 °C in Luria‐Bertani (LB) broth. Ampicillin was used at 50 µg mL^−1^, kanamycin at 50 µg mL^−1^, and chloramphenicol at 25 µg mL^−1^.

HAP1 cells, gift from the Taipale Lab (originally purchased from Horizon Discovery), were cultured in Iscove's Modified Dulbecco's Medium (IMDM) supplemented with 10% Fetal Bovine Serum (FBS) and 1% Penicillin–Streptomycin. INT407 cell line (ATCC No. CCL‐6) was cultured in Dulbecco's modified Eagle's medium (DMEM) supplemented with 10% Fetal Bovine Serum (FBS) and 1% Penicillin–Streptomycin. All cells were maintained at 37 °C in a humidified 5% CO_2_ incubator. Cultures were routinely tested for mycoplasma contamination using MycoStrip™ detection kits (InvivoGen).

### Lentiviral Library Production and Transduction

To conduct a genome‐wide knockout screen, the HAP1 cell line was transduced with lentiviruses containing the TKOv3 gRNA library pool. The lentivirus was produced by transfecting HEK293T packaging cells with the TKOv3 gRNA plasmid pool. Three days later, the lentivirus‐containing culture medium was collected and filtered through a 0.44 µm syringe filter. HAP1 cells were then treated with the collected virus at a MOI of 0.3. To ensure that all ≈70,000 gRNAs were represented, 50 million cells (200‐fold coverage) were infected. Infected cells were selected by puromycin for the following 3 days, and only the surviving cells were collected (T0).

### *Salmonella* Invasion Screen

The CRISPR knockout cell library was cultured and expanded until T5, ensuring a minimum library coverage of 100‐fold was maintained at each passage. On T7, cells were seeded into two 15 cm plates for each replicate. The following day (T8), cells were infected with GFP‐expressing *S*. Typhimurium at an MOI of 50. A parallel culture of uninfected cells was maintained as a control. At 21 h post‐infection, cells were detached using an enzyme‐free cell dissociation buffer (1 mm EDTA, 150 mm NaCl, 5 mm NaHCO_3_, 0.1% glucose, and 10 mm KCl) and subsequently sorted by FACS for GFP‐negative population. The invasion screen was conducted in triplicate. Sorted population was then replated in normal growth medium for expansion. Once the cells reached confluency, they were harvested along with the corresponding time‐matched uninfected control population for further analysis.

### Cell Fitness Screen

For the long‐term fitness screen, a parallel set of cells infected at T8 was cultured until T22. To eliminate extracellular bacteria and prevent re‐infection, the culture medium was supplemented with gentamycin. On T14 and T22, 7 million cells were harvested from both the infected and uninfected control populations. The harvested cells were pelleted and stored frozen for subsequent analysis.

### Next‐Generation Sequencing Library Preparation

To analyze the gRNA population of each sample, genomic DNA was extracted using the Wizard Genomic DNA Purification Kit (Promega) following the manufacturer's protocol. The gDNA was amplified in a two‐step PCR process. The first PCR was performed to amplify the gRNA region in each sample. The second PCR was conducted to amplify and barcode each sample. The completed gRNA library was sequenced on Illumina Next‐seq high‐output mode at a read depth of at least 7 million reads per sample. MAGeCK algorithm was used to generate rankings for positively enriched genes.

### Construction of Knockout Cell Lines

To construct a polyclonal knockout cell line, one sgRNAs targeting SORL1 and ZFYVE19 were designed and were cloned into pX459 (Addgene plasmid #62988). HAP1 and INT407 cells were seeded in a 6‐well plate at a density of 0.3 million cells per well. The following day, the cells were transfected with cloned plasmids, using the Lipofectamine 3000 reagent (Invitrogen). Twenty‐four hours after transfection, the cells were treated with puromycin (2.0, 1.5 µg µL^−1^) to select for successfully transfected cells.^[^ ^44^ ^]^

### KO Cell Line Validation

To assess the knockout efficiency of SORL1, total RNA was first extracted from the generated cell line. Following reverse transcription to synthesize cDNA, the relative abundance of SORL1 mRNA was determined by qPCR, with normalization to GAPDH expression.^[^ ^45^ ^]^

Western blot analysis was performed with cell lysates from HAP1, INT407 WT or KO cells were prepared under denaturing conditions by using anti‐SORL1 (1:1,000 dilution in TBST with 3% BSA, Cell Signaling Technology, 79322) and anti‐GAPDH (1:1,000 dilution in TBS with 3% BSA, Cell Signaling Technology, 2118). The blots were developed by incubation with ·anti‐rabbit IgG horseradish peroxidase‐linked antibodies (1:10,000 dilution, Invitrogen, 31 460) for 1 h, and were detected using the ECL detection system (Merck Millipore, WBULS0500).

The knockout efficiency of ZFYVE19 was examined using TIDE analysis.^[^ ^46^ ^]^ The genomic DNA region containing the gRNA sequence was amplified by PCR from genomic DNA extracted from the cells. The resulting PCR products were sequenced and the results from the transfected cells were compared with those from WT cells. This analysis was conducted using the program provided at tide.nki.nl to confirm the knockout efficiency.

To obtain a monoclonal cell line, a serial dilution method was performed with the polyclonal cell line. Cells were seeded in a 96‐well plate and serially diluted, and wells containing a single cell were selected. After expanding the single‐cell‐derived clones to confluency at 10‐cm plate, the mutation was confirmed by TIDE analysis as described above.

### Bacterial Infections for Validation

Briefly, 2.5 × 10^5^ HAP1 cells in IMDM supplemented with 10% FBS or 2.5 × 10^5^ INT407 cells in DMEM supplemented with 10% FBS were seeded in 24‐well plates and cultured at 37 °C in a humidified 5% CO_2_ incubator. For bacterial culture, *Salmonella* was grown overnight in LB medium and then subcultured at a 1/20 dilution in fresh LB medium and incubated with a shaking incubator at 37 °C for 3 h. Bacteria were added to the pre‐seeded cells at a multiplicity of infection (MOI) of 50. The plates were centrifuged at 1500 rpm for 5 min at room temperature and incubated for an additional 1 h. Then, the extracellular bacteria were washed three times with PBS (phosphate‐buffered saline) and killed by incubation with IMDM or DMEM supplemented with 10% FBS and 120 µg mL^−1^ gentamicin for 1 h. The media was replaced after 1 h with fresh IMDM or DMEM containing 12 µg mL^−1^ gentamicin and the incubation was continued at 37 °C.

To measure the number of bacteria that are attached, invaded, or replicated, cells were lysed with PBS containing 0.1% Triton X‐100 and plated on LB plates with appropriate dilutions. The percentage of bacterial attachment was determined by dividing the number of bacteria recovered from host cells before gentamicin treatment by the total number of host cells. The percentage of bacterial invasion and replication was determined by dividing the number of bacteria recovered from host cells at 1 h or 21 h after gentamicin treatment by the total number of host cells. All experiments were performed in triplicate.

### Flow Cytometry

Briefly, 1 × 10^6^ HAP1 cells in IMDM supplemented with 10% FBS were seeded in six‐well plates and cultured at 37 °C in a humidified 5% CO_2_ incubator. For bacterial culture, *Salmonella* harboring pFPV25.1 vector was grown overnight in LB medium and then subcultured at a 1/20 dilution in fresh LB medium and incubated with a shaking incubator at 37 °C for 3 h. Cells were then infected with *Salmonella* as described above.

At the indicated time points, infected cells were washed and detached using Cell Dissociation Buffer (Gibco). The fluorescence of bacteria inside cells was subsequently assessed by FACS analysis.^[^ ^47^ ^]^ Samples were analyzed on a NovoCyteTM Flow Cytometer (ACEA) using NovoExpress software (ACEA). On the NovoCyteTM Flow Cytometer, fluorophores were excited at a wavelength of 488 nm, with green fluorescence detected at 530 nm. Data were analyzed with NovoExpress software.

For measuring *Salmonella* invasion or replication inside cells, cells were gated using an FSC/SSC dot plot to select HAP1 cells, and GFP plots were analyzed to determine the MFIs of *Salmonella* within cells. *Salmonella* replication efficiency within cells was calculated as [MFI of GFP‐expressing *Salmonella* inside cells at 21 h post‐infection/ MFI of GFP‐expressing *Salmonella* inside cells at 1 h post‐infection]. All experiments were performed in triplicate and the results are representative of three independent experiments.

### Confocal Immunofluorescence Microscopy

For confocal imaging, sterilized round glass coverslips were placed in 24‐well plates. 1 × 10^5^ HAP1 cells were seeded onto the prepared coverslips and incubated overnight. Cells were then infected with *Salmonella* harboring the pFPV25.1 vector as described above. At specified time points post‐infection, cells on coverslips were fixed with 0.5 mL of 4% paraformaldehyde (PFA) for 20 min at room temperature. Fixed cells were washed three times with PBS. Permeabilization was performed by incubating cells with 0.1% Saponin (Sigma–Aldrich) in 1% BSA (Sigma–Aldrich) in PBS for 20 min. After removing the permeabilizing buffer, cells were blocked with 1% BSA in PBS for 20 min and coverslips were moved to a petri dish layered with parafilm. For separate experiments, cells were incubated with Tubulin Mouse mAb (1:1,000 dilution in 0.1% Saponin in PBS; ABclonal, AC012) in 100 µL drops per coverslip overnight at 4 °C and coverslips were washed with 0.1% Saponin in PBS. To detect the primary antibodies, cells were incubated for 1 h at room temperature in the dark with Alexa Fluor 647‐conjugated AffiniPure F(ab')_2_ Fragment Donkey Anti‐Mouse IgG (H+L) (1:1,000 dilution in 0.1% Saponin in PBS; Jackson ImmunoResearch, Cat# 715‐606‐151, RRID: AB_2340866). Coverslips were then washed two times with 0.1% Saponin in PBS (5 min each), followed by two washes with 1X PBS (5 min each). To visualize cell nuclei, cells were stained with DAPI (4′, 6‐diamidino‐2‐phenylindole) for 30 min in the dark. After two final washes with PBS, coverslips were mounted onto glass slides using an appropriate mounting agent (Sigma–Aldrich). Slides were allowed to dry in a dark place at room temperature before being stored in a slide box at 4 °C. Images were acquired using a confocal microscope. Z‐stack images were obtained spanning a total depth of 8 µm with 0.5 µm intervals, resulting in 17 slices.

### GTEx Data Analysis and Visualization

The data used for the analyses described in this manuscript were obtained from the GTEx Portal on 07/09/25. The analysis utilized the gene TPM (Transcripts Per Million) values file processed by RNA‐SeQC v2.4.2 and the corresponding sample metadata file. All data processing was performed within the R statistical environment. The expression row for the gene of interest (SORL1) was extracted from the full expression matrix. This gene expression data was then merged with the sample metadata based on SAMPID to map tissue information (SMTSD) to each sample's expression value. Data visualization was conducted using the ggplot2 package. Analysis was restricted to a pre‐selected subset of tissues, and a combined violin and box plot was generated to illustrate the distribution of raw TPM values.

### Antibody‐Mediated Blockade of SORL1

1 × 10^4^ HAP1 cells were seeded in 96‐well plates and incubated overnight. Prior to *Salmonella* infection, 8 µL of anti‐SORL1 antibody (BD, 611860) at a stock concentration of 250 µg mL^−1^ was added to each well (Final concentration of 20 µg mL^−1^) and incubated for 30 min. As controls, secondary anti‐mouse IgG (Cellnest, CNG004‐0005) and PTH1R (Invitrogen, PA3‐205) were used at the same concentration. Subsequently, 50 µL of *Salmonella* was added to each well at a MOI of 50, followed by a 30 min incubation. After 1 h of gentamicin treatment, cells were lysed with PBS containing 0.1% Triton X‐100 and plated on LB plates with appropriate dilutions. The percentage of bacterial invasion was determined by dividing the number of CFUs recovered from host cells by the total number of host cells. All experiments were performed in triplicate and the results are representative of three independent experiments.

### Cell Viability Assay

Cell viability was assessed using the Max‐Blue Resazurin Cell Viability Assay Kit (Biomax) by measuring fluorescence according to the manufacturer's instructions. Briefly, HAP1 cells were treated with antibodies and infected with *Salmonella* as described above. Max‐Blue reagent was added directly to each well (10% of the total volume) and incubated for 1 h at 37 °C. The fluorescence intensity was measured using a microplate reader with excitation at 530 nm and emission at 590 nm. The % of cell viability was calculated considering fluorescence value of antibody‐untreated HAP1 WT as 100%.

### Transient Transfections and Co‐Immunoprecipitation

HEK293T cells were transfected with plasmids expressing FLAG‐tagged ZFYVE19 or its variants and HA‐tagged VPS4A, VPS4B, CHMP4A, and CHMP4C. ≈1 × 10^6^ HEK293T cells in DMEM supplemented with 10% FBS were seeded in six‐well plates and transiently transfected after 24 h with expression plasmids by using by jetPRIME (Polyplus) according to the manufacturer's instructions. After 24 h post‐transfection, cells were scraped from the plate in 1× DPBS for sonication. A portion of this whole‐cell lysate was reserved as the input sample. The remaining lysate was incubated with EZview Red Anti‐HA Affinity Gel (Sigma–Aldrich) overnight at 4 °C on nutating mixer. Beads were subsequently washed five times with 1× TBS, and bound proteins were eluted by boiling in SDS‐PAGE loading buffer (Biosesang). Input and eluted samples were then heated at 95 °C for 10 min prior to SDS‐PAGE and western blotting analysis. Western blot analysis was performed with input and immunoprecipitated (IP) samples by using anti‐HA (1:5,000 dilution, Sigma–Aldrich) and anti‐FLAG (1:5,000 dilution, Sigma–Aldrich) antibodies. The blots were developed by incubation with anti‐rabbit IgG horseradish peroxidase‐linked antibodies (1:10,000 dilution, ThermoFisher) for 1 h, and were detected using the ECL detection system (SuperSignal West Femto Maximum Sensitivity Substrate, ThermoFisher).

### Construction of Plasmids

pDONR223 (Invitrogen) harboring cDNAs encoding ZFYVE19, VPS4A, VPS4B, CHMP4A, and CHMP4C were picked from human ORF collection. To obtain HA‐tagged or FLAG‐tagged proteins, these cDNAs were recombined using the gateway system into destination vectors pcDNA3.1‐ccdB‐3 × HA (Addgene plasmid #214986) or pcDNA3.1‐ccdB‐3 × FLAG‐V5 (Addgene plasmid #87063) with LR clonase II (Invitrogen).

### Construction of *Salmonella* SPI‐1 and SPI‐2 Deletion Mutant Strains

All primers used in this study are listed in Table S2 (Supporting Information). *S*. Typhimurium 14028s deletion strains, 𝛥SPI‐1::Km^R^ and 𝛥SPI‐2::Cm^R^, were generated by the one‐step gene inactivation method.^[^ ^48^ ^]^ A Km^R^ cassette for the SPI‐1 genes (*hilD*‐*invH*) was PCR amplified from plasmid pKD4 using primers 1013/937. A Cm^R^ cassette for the SPI‐2 genes (*sseB*‐*ssaU*) was PCR amplified from plasmid pKD3 using primers 1014/1002. The resulting PCR product was integrated into the 14028s chromosome to generate 𝛥SPI‐1 (𝛥SPI‐1::Km^R^) or 𝛥SPI‐2 (𝛥SPI‐2::Cm^R^).

### Statistical Analysis

All graphs were generated using Prism software (GraphPad). Unless otherwise stated, data are presented as the mean ± standard deviation (SD) of three independent experiments. Statistical significance was assessed using a one‐way ANOVA with Tukey's multiple comparisons test. A *p* value <0.05 was considered statistically significant.

## Conflict of Interest

The authors declare no conflict of interest.

## Author Contributions

S.Y., S.Y.K., and S.G.K. contributed equally to this work. H.L. and E.‐J.L. conceptualized and supervised the study, acquired funding, and were responsible for manuscript writing and revision. S.Y.K. and S.Y. designed and performed the CRISPR screens. S.Y. also performed all data interpretation and bioinformatics analysis and constructed the knockout cell lines. Most validation experiments, including bacterial infection assays, flow cytometry, and pull‐down assays, were performed by S.Y.K. and S.G.K. Western blot analysis of PTH1R expression was conducted by M.N. S.G.K. conducted all confocal microscopy and subsequent image quantification. The figures were designed by S.Y.K. and S.Y. The original manuscript was written by S.Y.K. and S.Y., and all authors contributed to the reading, editing, and approval of the final manuscript. D.‐K.K. shared the entry clones for the validation experiments and provided functional genomics expertise when designing the experimental scheme.

## Supporting information



Supporting Information

Supporting Information

## Data Availability

The data that support the findings of this study are available in the supplementary material of this article.
