# Supplementary material for: Parallel Genome‐Wide CRISPR Screens Reveal SORL1 and ZFYVE19 as Sequential Host Determinants of Salmonella Infection
Source: Adv Sci (Weinh). 2025 Nov 23;13(8):e15042. doi: 10.1002/advs.202515042 (PMC12884770; doi:10.1002/advs.202515042)
Supplement: Supplementary file 1 — Supporting Information [file ADVS-13-e15042-s002.pdf]

Supporting Information

Parallel Genome-wide CRISPR Screens Reveal SORL1 and ZFYVE19 as Sequential Host Determinants of *Salmonella* Infection

Sehee Yun[a,b+], Seoyeon Kim[a+], Seonggyu Kim[a+], Minsoo Noh[a,c], Dae-Kyum Kim[d,e], Eun-Jin Lee[a#], Hunsang Lee[a,b#]

[a] Department of Life Sciences, School of Life Sciences and Biotechnology, Korea University, Republic of Korea

[b] National Research Laboratory for Convergence Degradation Biology, Korea University, Republic of Korea

[c] Department of Internal Medicine and Laboratory of Genomics and Translational Medicine, Gachon University College of Medicine, Republic of Korea

[d] Division of Thoracic and Upper Gastrointestinal Surgery, Department of Surgery, Faculty of Medicine and Health Sciences, McGill University, Canada

[e] Cancer Research Program, Research Institute of McGill University Health Centre, Canada

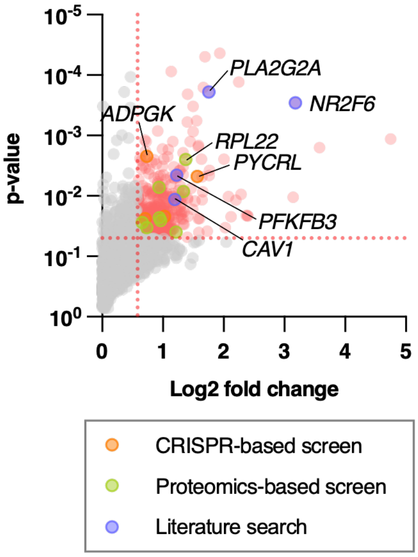

Supplemental Figure 1: Volcano plot of invasion screen using previous studies

Volcano plot of screen hits in invasion screen, highlighting overlaps with a previous CRISPR screen (orange) and AP-QMS data (green). Genes with known roles in *Salmonella* or bacterial infection are marked in blue.

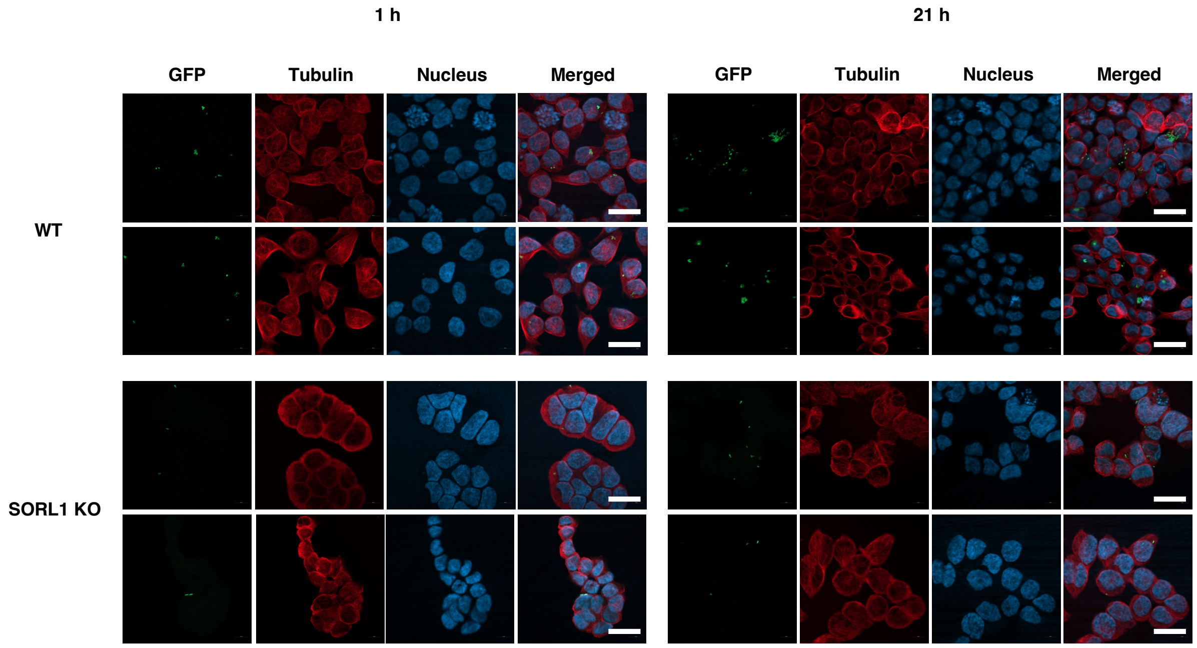

Supplemental Figure 2: Confocal images of *Salmonella*-infected HAP1 cells

Confocal microscopy images of HAP1 cells infected with GFP-expressing *Salmonella*. HAP1 WT or SORL1 KO cells were infected with GFP-expressing *Salmonella* and fixed at the indicated time points. *Salmonella* are visualized by GFP (green). Cells were stained for α-tubulin (red) and nuclei (blue). Merged images show the localization of *Salmonella* inside HAP1 cells. Scale bar, 50 µm.

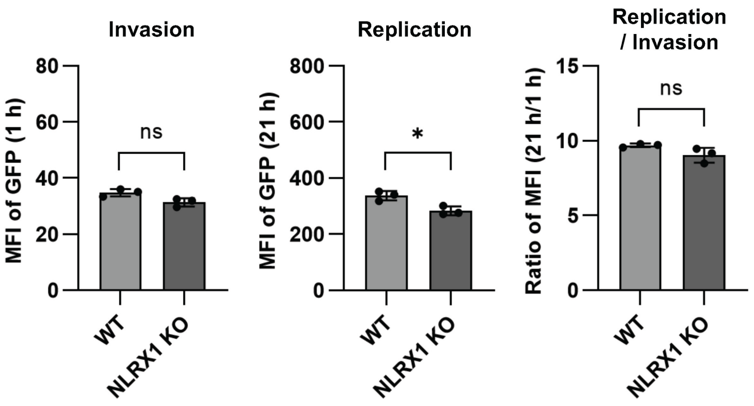

Supplemental Figure 3: NLRX1 has a minimal impact on the susceptibility to *Salmonella* infection

Flow cytometric analysis of bacterial load in HAP1 WT and NLRX1 KO cells at 1-hour (invasion) and 21-hours (replication) post-infection with GFP-expressing *Salmonella*. The ratio of MFI at 21 hours to 1 hour was also calculated as a metric for intracellular replication. Data represent mean ± SD (n = 3). p value ns; ≥0.5 and *; <0.05.

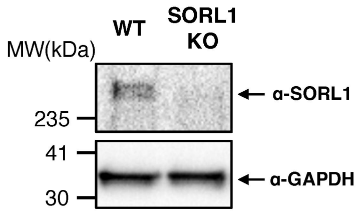

Supplemental Figure 4: INT407 SORL1 KO confirmation

Validation of SORL1 KO in INT407 cell lines by western blot analysis.

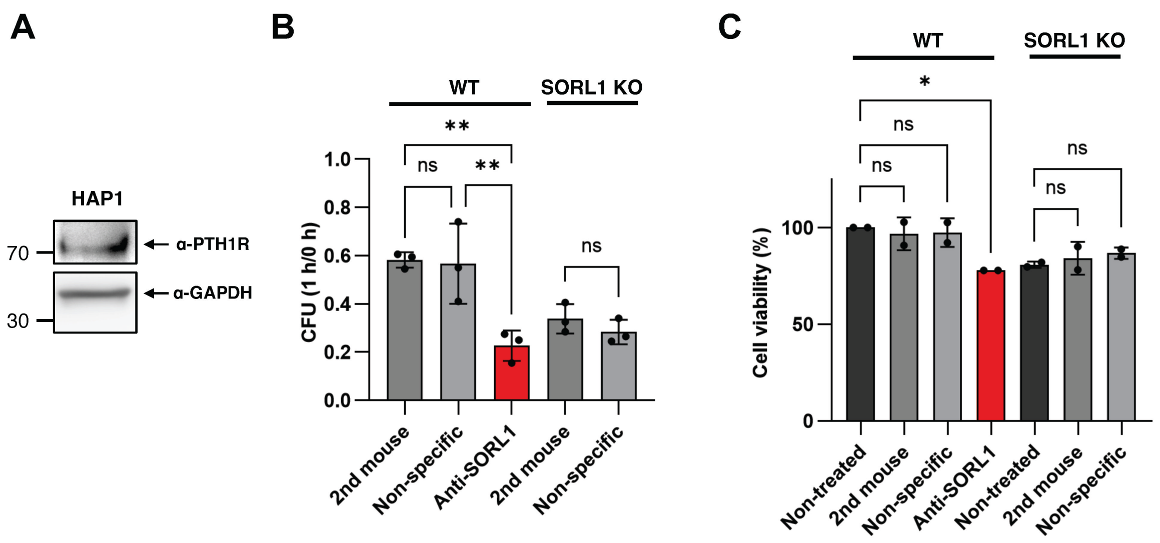

Supplemental Figure 5: Antibody-mediated inhibition of SORL1

(A) Western blot analysis showing PTH1R protein is expressed in HAP1 cells. **(B)** Fold invasion of *Salmonella* within HAP1 WT or SORL1 KO cells treated with anti-mouse (secondary mouse antibody), anti-PTH1R (non-specific control antibody), or anti-SORL1 antibody. Fold invasion represents [number of bacteria at 1 h / number of bacteria at 0 h]. This experiment was performed using an independent batch of cells and was not merged with the data shown in Figure 4C. Data represent mean ± SD (n = 3). p value ns; ≥0.5 and **; <0.01. **(C)** Determination of cell viability. The % of cell viability was calculated considering antibody-untreated HAP1 WT as 100% and plotted. Data represent mean ± SD (n = 3). p value ns; ≥0.5 and *; <0.05.

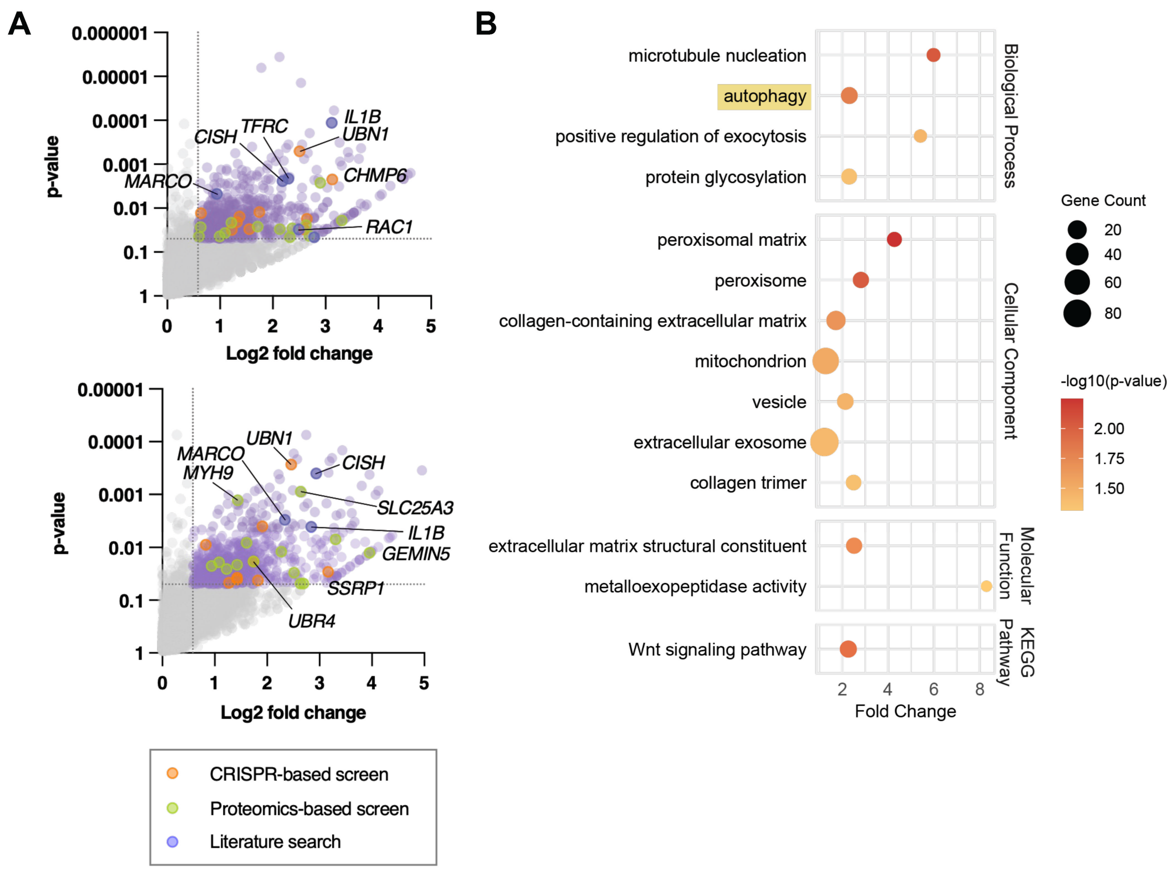

Supplemental Figure 6: Validation of fitness screen using previous studies

(A) Volcano plot of screen hits in fitness screens, highlighting overlaps with a previous CRISPR screen (orange) and AP-QMS data (green). Genes with known roles in *Salmonella* or bacterial infection are marked in blue. **(B)** GO enrichment analysis of depleted genes identified at the day 14 time point. Each dot represents an enriched biological term. The fold enrichment is plotted on the x-axis, the p-value is indicated by the dot's color, and the number of genes associated with each term is represented by the dot's size.

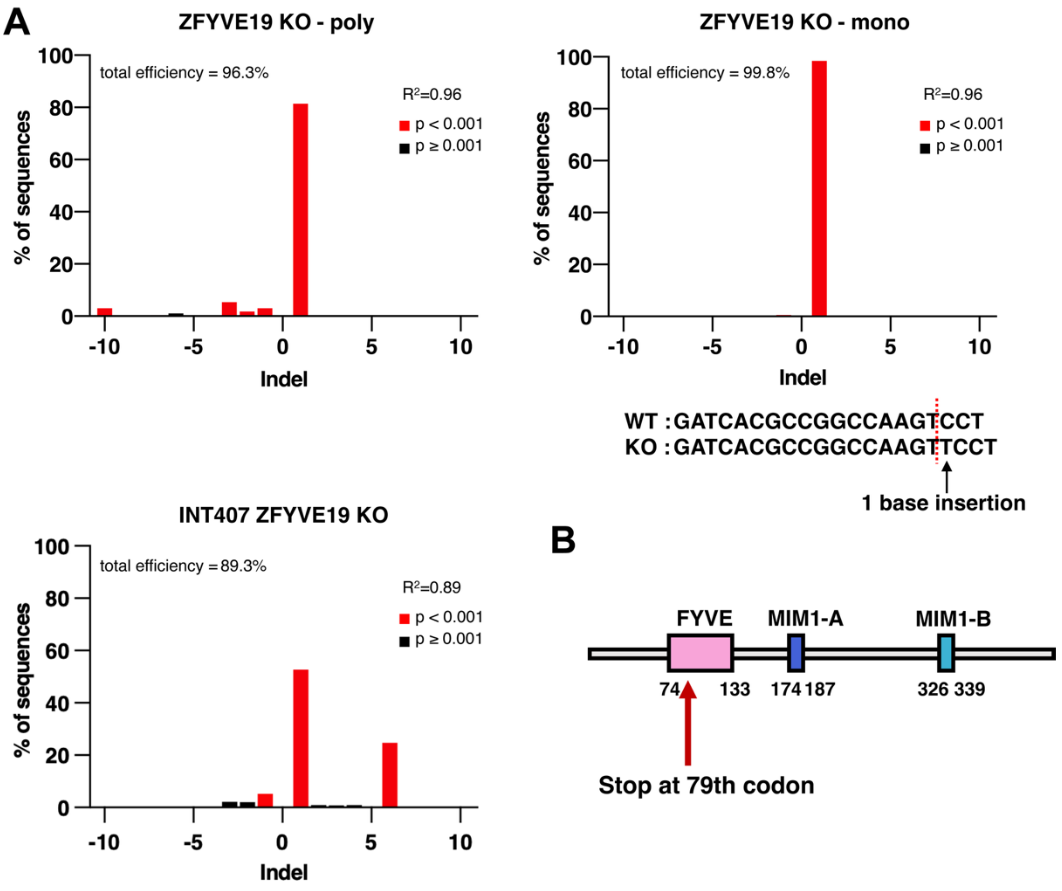

Supplemental Figure 7: Confirmation of ZFYVE19 knockout efficiency by TIDE analysis

(A) A bar graph quantifying the editing efficiency in the generated ZFYVE19 KO cell lines. The y-axis represents the total frequency (%) of insertions and deletions (indels) at the target locus, as determined by TIDE analysis of Sanger sequencing chromatograms. The specific indel sequence identified in the validated monoclonal cell line is annotated beyond its corresponding graph. **(B)** Schematic diagram illustrates how these indels result in frameshifts and premature stop codons.

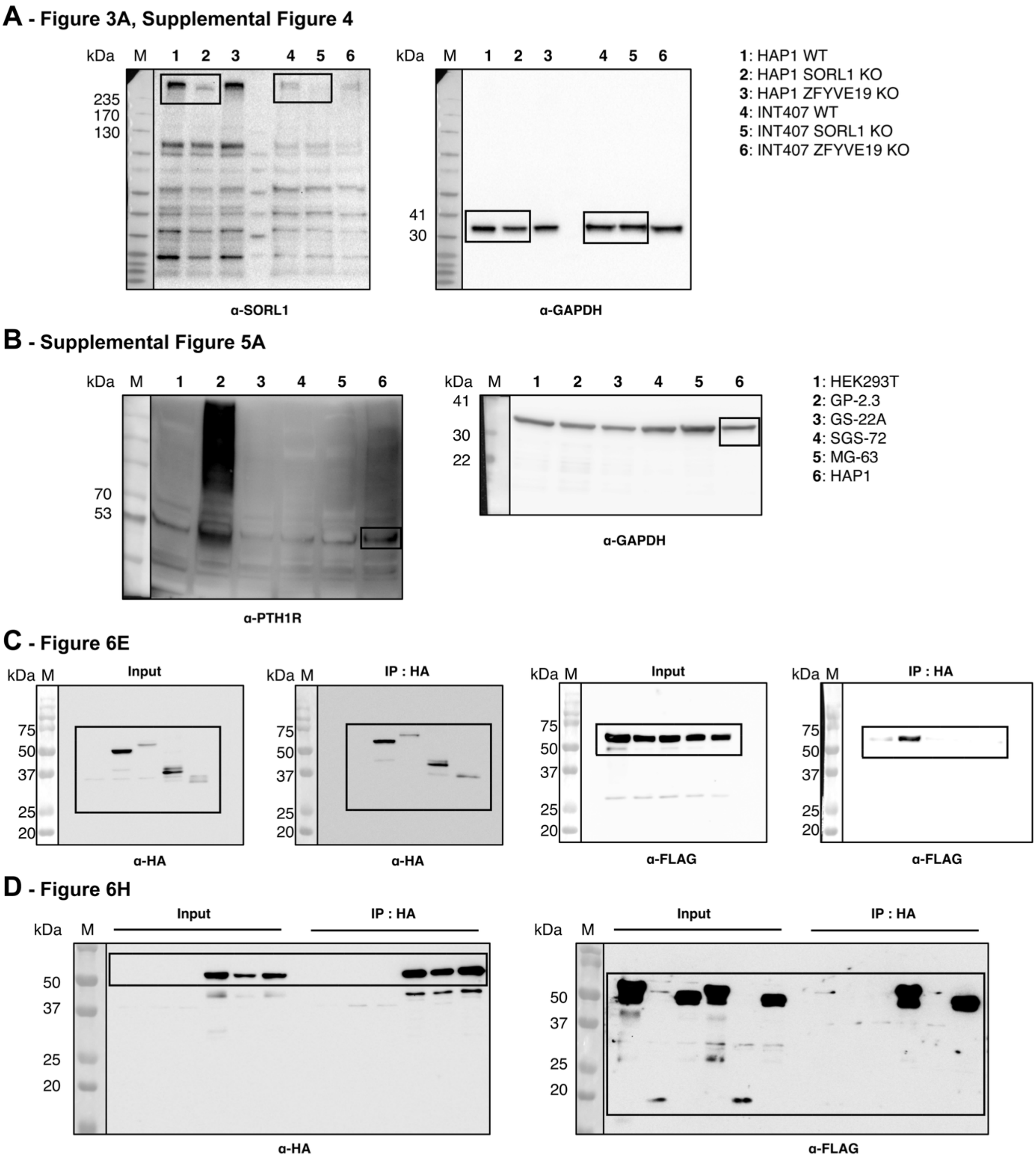

Supplemental Figure 8. Unedited western blot images

Uncropped images of western blots displayed in Figure 3A, Supplemental Figure 4 **(A)**, Supplemental Figure 5A **(B)**, Figure 6E **(C)**, and Figure 6H **(D)**. Molecular weight markers are indicated. The specific regions used in the main figures are outlined with dashed lines. Molecular weight markers were imaged using epi-white light, while target protein bands were developed by treating the membranes with a chemiluminescent substrate solution.

Supplemental Table 1: Bacterial strains and plasmids used in this study

Name	Description	Reference
***S. enterica* serovar Typhimurium**		
14028s	wild-type	[1]
SY244	14028s/pFPV25.1	This study
SY488	*Δ*SPI-1(*hilD-invH*::Km^R)	This study
SY489	*Δ*SPI-2(*sseB-ssaU*::Cm^R)	This study
Escherichia coli		
DH5α	*fhuA2 lac(del)U169 phoA glnV44 Φ80' lacZ(del)M15 gyrA96 recA1 relA1 endA1 thi-1 hsdR17*.	[2]
SY430	DH5α/pcDNA3.1-ZFYVE19-3XFLAG	This study
SY431	DH5α/pcDNA3.1-ZFYVE19_FYVE-3XFLAG	This study
SY432	DH5α/pcDNA3.1-ZFYVE19_MIM1-3XFLAG	This study
SY433	DH5α/pcDNA3.1-VPS4A-3XHA	This study
SY434	DH5α/pcDNA3.1-VPS4B-3XHA	This study
SY435	DH5α/pcDNA3.1-CHMP4A-3XHA	This study
SY436	DH5α/pcDNA3.1-CHMP4C-3XHA	This study
**Plasmids**		
pFPV25.1	rep_{pMB1} Ap^R, promoterless *gfp*	[3]
pKD3	$repR_{6K\gamma}$ Ap^R FRT Cm^R FRT	[4]
pKD4	$repR_{6K\gamma}$ Ap^R FRT Km^R FRT	[4]
pKD46	rep_{pSC101}^{ts} Ap^R P_{araBAD} γ β exo	[4]
pcDNA3.1-3XFLAG	pcDNA3.1-ccdB-3xFLAG-V5	[5]
pcDNA3.1-3XHA	pcDNA3.1-ccdB-3xHA-V5	
pX459I	pSpCas9(BB)-2A-Puro	[6]

Supplemental Table 2: Primers used in this study

Name	Sequence (from 5' to 3')*	
SZ188	GGGGACAACTTTGTACAAAAAAGTTGGCATGGAG AGTAGGTGCTACGG	ZFYVE19 FYVE domain cloning
SZ189	GGGGACAACTTTGTACAAGAAAGTTGGGTATGGT GACCACTTGGAGGC	ZFYVE19 FYVE domain cloning
SZ190	GGGGACAACTTTGTACAAAAAAGTTGGCATGCCA CCTCAGAACTATAAG	ZFYVE19 MIM1 domain cloning
SZ191	GGGGACAACTTTGTACAAGAAAGTTGGGTAGTGC TCTTGGCCTGCA	ZFYVE19 MIM1 domain cloning
1013	GCTGGAAGGATTTCCTCTGGCAGGCAACCTTATA ATTTCA TGTAGGCTGGAGCTGCTTCG	SPI-1 deletion
937	TAATTATATCATGATGAGTTCAGCCAACGGTGATAT GGCCCATATGAATATCCTCCTTAG	SPI-1 deletion
1014	CAAAATATGACCAATGCTTAATACCATCGGACGCC CCTGG TGTAGGCTGGAGCTGCTTCG	SPI-2 deletion
1002	CAACGGGTTCAAATAACGTTTCAGGAATTTTATCT CCGCG CATATGAATATCCTCCTTAG	SPI-2 deletion
1015	GCAAGGTTAACACTTTTTTGCCTGC	SPI-1 confirmation
1015-2	GCGAGGTGCTGCTGTATCAAAAAG	SPI-1 confirmation
1016	AATGAAGACTAAGGTAGTGATAAATG	SPI-2 confirmation
1016-2	GCTCTATACGCTATTACCCTC	SPI-2 confirmation
Z1_F	CCTCTCAAGATCAGCCTTCCTC	TIDE primer for ZFYVE19 KO validation
Z1_R	AGAAAAGAGTAGCTTTGCAAAAACG	TIDE primer for ZFYVE19 KO validation
SQ_F	CCACCAACCGAAGACGTGTA	qPCR primer of SORL1
SQ_R	GCAAGTCAAGGTGCTGTGTG	qPCR primer of SORL1
GQ_F	GTGGACCTGACCTGCCGTCT	qPCR primer of GAPDH
GQ_R	GGAGGAGTGGGTGTCGCTGT	qPCR primer of GAPDH

Supplemental Table 3: gRNA sequences used in this study

Name	Sequence (from 5' to 3')*	
SORL1_sgRNA	GTGTACGTGTCTTACGACTA	SORL1 knockout
ZFYVE19_sgRNA	GATCACGCCGGCCAAGTCCT	ZFYVE19 knockout

Supplemental References

1. Fields, P.I., et al., *Mutants of Salmonella typhimurium that cannot survive within the macrophage are avirulent.* Proc Natl Acad Sci U S A, 1986. **83**(14): p. 5189-93.
2. Taylor, R.G., D.C. Walker, and R.R. Mcinnes, *Escherichia-Coli Host Strains Significantly Affect the Quality of Small-Scale Plasmid DNA Preparations Used for Sequencing.* Nucleic Acids Research, 1993. **21**(7): p. 1677-1678.
3. Valdivia, R.H. and S. Falkow, *Bacterial genetics by flow cytometry: rapid isolation of Salmonella typhimurium acid-inducible promoters by differential fluorescence induction.* Mol Microbiol, 1996. **22**(2): p. 367-78.
4. Datsenko, K.A. and B.L. Wanner, *One-step inactivation of chromosomal genes in Escherichia coli K-12 using PCR products.* Proc Natl Acad Sci U S A, 2000. **97**(12): p. 6640-5.
5. Taipale, M., et al., *Quantitative analysis of HSP90-client interactions reveals principles of substrate recognition.* Cell, 2012. **150**(5): p. 987-1001.
6. Ran, F.A., et al., *Genome engineering using the CRISPR-Cas9 system.* Nat Protoc, 2013. **8**(11): p. 2281-2308.
